# Supplementary material for: Economic costs of severe seasonal influenza in Colombia, 2017–2019: A multi-center analysis
Source: PLoS One. 2022 Jun 17;17(6):e0270086. doi: 10.1371/journal.pone.0270086 (PMC9205505; doi:10.1371/journal.pone.0270086)
Supplement: S1 Table — (DOCX) [file pone.0270086.s001.docx]

**S1 Table. Items included in the direct cost of hospitalized influenza cases**

| **Category** | **Item description** |
| --- | --- |
| Hospital length of stay (LOS) | How long the patient lasts in the care services (hours or days). Include cost of the accompanying bed was included in this item. |
| Diagnostic and laboratory tests | Diagnostic images: X-rays, ultrasonography (abdomen, pelvis, thorax, urinary tract, brain), echocardiogram, doppler, computed axial tomography.  Clinical Laboratory: Blood count, urine analysis, detection of influenza by IFA or PCR, blood chemistry tests (urea nitrogen, creatinine, chlorine, glucose, electrolytes, transaminases, bilirubins) glucometry, immunological tests (c-reactive protein, antibody detection for pathologies such as dengue), hematological tests (cross-tests, coagulation times, reticulocyte count, hemoglobin electrophoresis, blood cultures, processing and transfusion of blood products, indirect Coombs, antibody screening), hormone determination, arterial gases, cultures (blood cultures, urine cultures, co-cultures), Gram staining and reading of any sample, determination of drugs of abuse or psychoactive substances (opiates, amphetamines, cocaine) or determination of drug levels. |
| Medications | Salbutamol, acetaminophen, furosemide, ampicillin, adrenaline, antibiotics, dipyrone, tramadol, heparin, fentanyl, lidocaine among others; as well as master preparations, oxygen, nutritional support, breast formula milk and intravenous solutions or fluids used in fluid therapy. |
| Consultations | General medicine, consultations by specialist (pediatrics, pediatric hemato-oncology, internal medicine, hematology, anesthesiology, surgery, psychiatry, physical medicine, maternal fetal, infectiology, emegency, gynecology and obstetrics), psychology and nutrition. Also, physical, respiratory, speech, and occupational therapies. |
| Procedures | Fibrobronchoscopy, blood components transfusion, nebulization, oximetry, autopsy, electrocardiogram, colonoscopy |
| Supplies | Sterile water, buretrol, extension equipment, needles, syringes, inhalo-chamber, gauze, connector, catheter, nasal cannula, humidifier, macro-drip and micro-drip equipment, probe, tube suction, infusion tubes, pressure valves, hose, dressings, gloves, venoclysis equipment, suture material, mask, electrodes, colostomy barrier. |
