# Supplementary material for: Economic costs of severe seasonal influenza in Colombia, 2017–2019: A multi-center analysis
Source: PLoS One. 2022 Jun 17;17(6):e0270086. doi: 10.1371/journal.pone.0270086 (PMC9205505; doi:10.1371/journal.pone.0270086)
Supplement: S2 Table — (DOCX) [file pone.0270086.s002.docx]

S2 Table. Comorbidities in the sample of direct costs

| Comorbidities | Number of patients |
| --- | --- |
| Chronic lower respiratory diseases | 20 |
| Circulatory system diseases | 7 |
| Neoplasias | 5 |
| genitourinary system Diseases | 4 |
| Moderate and mild protein-calorie malnutrition | 3 |
| Certain conditions originating in the perinatal period | 2 |
| Nervous System Diseases | 2 |
| Endocrine, nutritional and metabolic diseases | 2 |
| Other disorders of glucose regulation and internal secretion of the pancreas | 2 |
| Human immundeficiency virus [HIV] disease | 1 |
| Diseases of the eye and its annexes | 1 |
| Diseases of the blood and hematopoietic organs, and of certain disorders that affect the mechanism of immunity | 1 |
| Infections with predominantly sexual transmission mode | 1 |
| Congenital malformations, deformities, and chromosomal abnormalities | 1 |
| Transplanted organs and tissues | 1 |
| Other pleural diseases | 1 |
| Mental and behavioral disorders | 1 |
| Total | 55 |
