# Supplementary material for: Economic costs of severe seasonal influenza in Colombia, 2017–2019: A multi-center analysis
Source: PLoS One. 2022 Jun 17;17(6):e0270086. doi: 10.1371/journal.pone.0270086 (PMC9205505; doi:10.1371/journal.pone.0270086)
Supplement: S3 Table — (DOCX) [file pone.0270086.s003.docx]

S3 Table. Characteristics people´s surveyed: indirect costs and out-of-pocket expenses

| **Variable** | **%** |
| --- | --- |
| **Educational level** | |
| Incomplete elementary school | 9.09 |
| Secondary incomplete | 13.64 |
| Secondary complete | 13.64 |
| Professional | 4.55 |
| Not reported | 59.09 |
| **Occupation** | |
| Student | 13.64 |
| Housewife | 18.18 |
| Dependent worker | 4.55 |
| Independent worker | 4.55 |
| Does not work | 13.64 |
| Not reported | 45.45 |
| **Number of people in the household** |  |
| <5 | 77.27 |
| 6 a 10 | 13.64 |
| 11 a 15 | 9.09 |
| **People contribute financially to the household** | |
| Mother and Father | 27.27 |
| Partner | 13.64 |
| Patient | 9.09 |
| Father | 9.09 |
| Mother | 4.55 |
| Partner and Other | 9.09 |
| Patient and Other | 9.09 |
| Mother and Other | 9.09 |
| Father and Other | 9.09 |
| **Range the patient's monthly income (in minimum wages)** | |
| N.A. (For childs) | 50.00 |
| Less than U$ 240.4 | 4.55 |
| Between U$ 240.4 and less than U$ 480.8 | 22.73 |
| Between U$ 480.8 and less than  U$ 721.2 | 9.09 |
| Between U$ 721.2 and less than  U$ 961.6 | 4.55 |
| More than U$ 1,202.0 | 9.09 |
| **Range the monthly household income (in minimum wages)** | |
| N.A. (For childs) | 4.55 |
| Less than U$ 240.4 | 13.64 |
| Between U$ 240.4 and less than U$ 480.8 | 36.36 |
| Between U$ 480.8 and less than  U$ 721.2 | 13.64 |
| Between U$ 721.2 and less than  U$ 961.6 | 13.64 |
| Between U$ 961.6 and U$ 1,202.0 | 9.09 |
| More than U$ 1,202.0 | 9.09 |
