# Supplementary material for: Economic costs of severe seasonal influenza in Colombia, 2017–2019: A multi-center analysis
Source: PLoS One. 2022 Jun 17;17(6):e0270086. doi: 10.1371/journal.pone.0270086 (PMC9205505; doi:10.1371/journal.pone.0270086)
Supplement: S1 Tool — (DOCX) [file pone.0270086.s004.docx]

**DIRECT COSTS SURVEY IN THE FRAMEWORK OF SURVEILLANCE OF ARI IN COLOMBIA, 2018**

CONFIDENTIAL: The data requested in this form is confidential. we will be used only for analysis purposes and individual information will not be published.

| 1 date of completion: DD/MM/YYYY | 2. Survey ID: | 3 Survey number: |
| --- | --- | --- |
| 4. City: | 5. Hospital / Clinic: | |
| **IDENTIFICATION AND SOCIECONOMIC DATA OF THE PATIENT** | | |
| 6. Type ID: CC___ TI____ CE___ | 7. ID: ___________________ | 8. Clinical Record ID ______________ |
| 9. Date of birth: DD/MM/YYYY | 10. Age fulfilled: ______________ | 11. Sex: M ______ F_______ |
| 12. If you are a woman, do you pregnant?  Yes__ No__ | 13. CIE-X code entry ____________ | 14. ICD-X code Hospital discharge ___________ |
| 15. Final condition  Alife _____ dead ______ | 16. City of residence | 17. Area: rural______ urban_____ |
| 18. Educational level:   \| Incomplete elementary school \|  \| \| --- \| --- \| \| Primary complete \|  \| \| Secondary incomplete \|  \| \| Secondary complete \|  \| \| Technician \|  \| \| Technologist \|  \| \| Professional \|  \| \| Postgraduate \|  \| \| N. A \|  \| | 19. You are affiliated with the General System of Social Security in Health  Yes_____ No_____  20. Regime of affiliation:   \| Contributory regime \|  \| \| --- \| --- \| \| Subsidized regime \|  \| \| Poor Uninsured Population \|  \| | 21. You have any of the following comorbidities   \| Asthma \|  \| \| --- \| --- \| \| Neurological \|  \| \| Autoimmune \|  \| \| COPD \|  \| \| Cardiovascular \|  \| \| Diabetes \|  \| \| Liver disorders \|  \| \| Renal disorders \|  \| \| Immunodeficiencies \|  \| \| Other \|  \|   Which one? ___________________ |
| **COST INFORMATION** | | |
| **EMERGENCIES** Days of stay___________  Laboratory tests and radiology (Including the diagnostic test for influenza)   \| **Name of the analysis: Diagnostic images and laboratory** \| **Number of times performed** \| **Unit cost** \| **Total cost** \| \| --- \| --- \| --- \| --- \| \|  \|  \|  \|  \| \|  \|  \|  \|  \| \|  \|  \|  \|  \| \|  \|  \|  \|  \| \|  \|  \|  \|  \| \|  \|  \|  \|  \| | | |
| \| **Name** \| **Route (oral, IV, inhalation etc)** \| **dose / day** \| **Dose unit (mg, ml etc)** \| **Administration frequency (single, once / day….)** \| **number of days** \| **Unit cost** \| **Total cost** \| \| --- \| --- \| --- \| --- \| --- \| --- \| --- \| --- \| \|  \|  \|  \|  \|  \|  \|  \|  \| \|  \|  \|  \|  \|  \|  \|  \|  \| \|  \|  \|  \|  \|  \|  \|  \|  \|   **Use of medicines and supplies.** | | |
| **Medical interconsultation**   \| **Medical Specialty** \| **Number of times performed** \| **Unit cost** \| **Total cost** \| \| --- \| --- \| --- \| --- \| \|  \|  \|  \|  \| \|  \|  \|  \|  \| \|  \|  \|  \|  \| | | |
| **Procedure**   \| **Medical Specialty** \| **Number of times performed** \| **Unit cost** \| **Total cost** \| \| --- \| --- \| --- \| --- \| \|  \|  \|  \|  \| \|  \|  \|  \|  \| \|  \|  \|  \|  \| | | |
| **HOSPITALIZATION** Days of stay___________  Laboratory tests and radiology (Including the diagnostic test for influenza)   \| **Name of the analysis: Diagnostic images and laboratory** \| **Number of times performed** \| **Unit cost** \| **Total cost** \| \| --- \| --- \| --- \| --- \| \|  \|  \|  \|  \| \|  \|  \|  \|  \| \|  \|  \|  \|  \| \|  \|  \|  \|  \| \|  \|  \|  \|  \| \|  \|  \|  \|  \| \|  \|  \|  \|  \| | | |
| \| **Name** \| **Route (oral, IV, inhalation etc)** \| **dose / day** \| **Dose unit (mg, ml etc)** \| **Administration frequency (single, once / day….)** \| **number of days** \| **Unit cost** \| **Total cost** \| \| --- \| --- \| --- \| --- \| --- \| --- \| --- \| --- \| \|  \|  \|  \|  \|  \|  \|  \|  \| \|  \|  \|  \|  \|  \|  \|  \|  \| \|  \|  \|  \|  \|  \|  \|  \|  \|   **Use of medicines and supplies.** | | |
| **Medical interconsultation**   \| **Medical Specialty** \| **Number of times performed** \| **Unit cost** \| **Total cost** \| \| --- \| --- \| --- \| --- \| \|  \|  \|  \|  \| \|  \|  \|  \|  \| \|  \|  \|  \|  \| | | |
| **Procedure**   \| **Medical Specialty** \| **Number of times performed** \| **Unit cost** \| **Total cost** \| \| --- \| --- \| --- \| --- \| \|  \|  \|  \|  \| \|  \|  \|  \|  \| \|  \|  \|  \|  \| | | |
| **INTENSIVE CARE UNIT (ICU) Days of stay___________**  **Laboratory tests and radiology (Including the diagnostic test for influenza)**   \| Name of the analysis: Diagnostic images and laboratory \| Number of times performed \| Unit cost \| Total cost \| \| --- \| --- \| --- \| --- \| \|  \|  \|  \|  \| \|  \|  \|  \|  \| \|  \|  \|  \|  \| | | |
| \| **Name** \| **Route (oral, IV, inhalation, etc)** \| **dose / day** \| **Dose unit (mg, ml, etc)** \| **Administration frequency (single, once / day….)** \| **number of days** \| **Unit cost** \| **Total cost** \| \| --- \| --- \| --- \| --- \| --- \| --- \| --- \| --- \| \|  \|  \|  \|  \|  \|  \|  \|  \| \|  \|  \|  \|  \|  \|  \|  \|  \| \|  \|  \|  \|  \|  \|  \|  \|  \|   **Use of medicines and supplies.** | | |
| **Medical interconsultation**   \| **Medical Specialty** \| **Number of times performed** \| **Unit cost** \| **Total cost** \| \| --- \| --- \| --- \| --- \| \|  \|  \|  \|  \| \|  \|  \|  \|  \| \|  \|  \|  \|  \| | | |
| **Procedure**   \| **Medical Specialty** \| **Number of times performed** \| **Unit cost** \| **Total cost** \| \| --- \| --- \| --- \| --- \| \|  \|  \|  \|  \| \|  \|  \|  \|  \| | | |
