# Supplementary material for: Economic costs of severe seasonal influenza in Colombia, 2017–2019: A multi-center analysis
Source: PLoS One. 2022 Jun 17;17(6):e0270086. doi: 10.1371/journal.pone.0270086 (PMC9205505; doi:10.1371/journal.pone.0270086)
Supplement: S2 Tool — (DOCX) [file pone.0270086.s005.docx]

**SURVEY OF INDIRECT COSTS AND POCKET EXPENSES IN THE FRAMEWORK OF**

**ARI SURVEILLANCE IN COLOMBIA**

**Date *RE-CONTACT*:** DD/MM/YYYY

| 1. Date: DD/MM/YYYY | | | 2. Survey ID: | | | | 3 Survey number: | |  |
| --- | --- | --- | --- | --- | --- | --- | --- | --- | --- |
| 4. City: | | | 5. Hospital / Clinic: | | | | 6. Phone: | |  |
| **IDENTIFICATION AND SOCIECONOMIC DATA OF THE PATIENT** | | | | | | | | |  |
| 7. Type ID: CC___ TI____ CE___ | | | 8. ID: ___________________ | | | | 9. Patient Medical Record ID: ____________ | |  |
| 10. Date of birth: DD/MM/YYYY | | | 11. Age: ______________ | | | | 12. Sex: M ______ F_______ | |  |
| 13. Area: rural______ urban_____  14. If you are a woman, do you pregnant?  Yes__ No__ | | | 15. ¿Are you affiliated to General Health System? Yes_______ No______  16. Regime of affiliation:  Contributory regime _____ Subsidized regime ____ Poor Uninsured Population ___ | | | | | |  |
| 17. Educational level:   \| Incomplete elementary school \|  \| \| --- \| --- \| \| Primary complete \|  \| \| Secondary incomplete \|  \| \| Secondary complete \|  \| \| Technician \|  \| \| Technologist \|  \| \| Professional \|  \| \| Postgraduate \|  \| \| N. A \|  \| | | | 18. Occupation:   \| Student \|  \| \| --- \| --- \| \| Housewife \|  \| \| Independent worker \|  \| \| Dependent worker \|  \| \| Does not work \|  \| \| N.A. \|  \| | | | | 19. Including yourself, how many people live in your household? Include children and adults: _________  20. Of these, how many contribute financially to the household? ___________ 20.1. Who is it?   \| Patient \|  \| \| --- \| --- \| \| Mother \|  \| \| Dad \|  \| \| Brother or Sister \|  \| \| Spouse \|  \| \| Other \|  \| | |  |
| 21. What range is the patient's monthly income? (minimal income)   \|  \| Less than U$ 240.4 \| \| --- \| --- \| \|  \| Between U$ 240.4 and less than U$ 480.8 \| \|  \| Between U$ 480.8 and less than  U$ 721.2 \| \|  \| Between U$ 721.2 and less than  U$ 961.6 \| \|  \| Between U$ 961.6 and U$ 1,202.0 \| \|  \| More than U$ 1,202.0 \| \|  \| N.A. (For children) \| | | | 22. What range is the monthly household income? (minimal income)   \|  \| Less than U$ 240.4 \| \| --- \| --- \| \|  \| Between U$ 240.4 and less than U$ 480.8 \| \|  \| Between U$ 480.8 and less than  U$ 721.2 \| \|  \| Between U$ 721.2 and less than  U$ 961.6 \| \|  \| Between U$ 961.6 and U$ 1,202.0 \| \|  \| More than U$ 1,202.0 \| | | | 23. What is the amount of the MONTHLY expenses of your household for the following items?   \| **Item** \| **Cost $** \| \| --- \| --- \| \| Food \|  \| \| Education \|  \| \| Rent \|  \| \| Home maintenance \|  \| \| Health \|  \| \| TOTAL \|  \| | | |  |
| **POCKET EXPENSES BEFORE HOSPITALIZATION** | | | | | | | | |  |
| 24. How many days have you had symptoms at the time of the consultation? ____________  25. Due to your illness, have you missed days from work or study?  Yes____ No _______  25.1 ¿How many? _____ | | 26. ¿How long take to transfer the patient to this institution including travel time and waiting time for transportation?   \| Less than half an hour \|  \| \| --- \| --- \| \| Between half an hour and 1 hour \|  \| \| Between 1 hour and 1 hour and ½ \|  \| \| Between 1 hour and ½ and 2 hours \|  \| \| More than 2 hours \|  \|   27. ¿How much the patient or his companion have to pay for transportation to this institution?  U$ _________________ | | | | | | 28. ¿Before entering this institution, did you receive any other type of care?  Yes_______ No_________  28.1. If yes, what type of care did she receive   \| **Item** \| Which one? \| # times \| \| --- \| --- \| --- \| \| Hospital or clinic \|  \|  \| \| Private doctor \|  \|  \| \| Traditional doctor \|  \|  \| \| Clinic \|  \|  \| \| Self-medication \|  \|  \| \| Other \|  \|  \| |  |
| \| **EXPENDITURE** \| **Hospital or clinic** \| **Private doctor** \| **Health center** \| **Traditional Medical** \| **Self-medication** \| \| --- \| --- \| --- \| --- \| --- \| --- \| \| Medicines \|  \|  \|  \|  \|  \| \| Laboratory tests \|  \|  \|  \|  \|  \| \| Diagnostic images (Rx) \|  \|  \|  \|  \|  \| \| Consultation \|  \|  \|  \|  \|  \| \| Transport \|  \|  \|  \|  \|  \| \| Copayment \|  \|  \|  \|  \|  \| \| Caregiver \|  \|  \|  \|  \|  \| \| Others \|  \|  \|  \|  \|  \|   29. Specify how much you had to pay for that prior care | | | | | | | | |  |
| **POCKET EXPENSES DURING HOSPITALIZATION** | | | | | | | | |  |
| 30. ¿How many days were you hospitalized? ______  31. During the hospitalization, did you have to pay for any concept  Yes_________ No______  31.1. If yes, which concept? | \| **Item** \| **Which one?** \| **Cost U$** \| \| --- \| --- \| --- \| \| Supplies \|  \|  \| \| Copayment \|  \|  \| \| Others (photocopies, food supplement) \|  \|  \|   32. ¿How many times did the caregiver or someone from the home travel to visit the patient? ___________  32.1. ¿What was the cost of each trip?  U$_____________________________ | | | | | | | 33. ¿Was necessary to hire a caregiver?  Yes____ No _______  33.1. ¿How much money did you pay? $___________________________  33.3 Did the payment to the caregiver affect the household finances? Yes____ No _____ |  |
| **PRODUCTIVITY LOSS** | | | | | | | | |  |
| 34. ¿In total, how long did the disease last from the onset of symptoms to its full resolution? ________ | | | | | 37. ¿You have stopped receiving income for the days or hours you do not work?  Yes____ No _______ 37.1 ¿How many? _____ | | | |  |
| 35. ¿Did you miss days of work or study because of your illness?  Yes____ No _______ 35.1 ¿How many? _____ | | | | | 38. ¿ Your caregiver or caregivers stopped receiving income for the time they dedicated to your care? Yes____ No _______ | | | |  |
| 36. ¿Did your caregivers loss study, working or activities days due to your disease?  Yes___ No ___ 36.1 ¿How many? _____ | | | | | 39. ¿How much money did they stop receiving? U$_________________ | | | |  |
| **POCKET EXPENSES AFTER HOSPITALIZATION** | | | | | | | | |  |
| 40. Type ID: CC__ TI__ CE__ 41. ID: _________________ 42. ¿How much did you spend on transportation the day you left hospitalization? __________ | | | | | | | | |  |
| 43. ¿Was it necessary to re-consult for this same disease? Yes____ No _______   \| **Ítem** \| Which one? \| Cost U$ \| \| --- \| --- \| --- \| \| Hospital or clinic \|  \|  \| \| Private doctor \|  \|  \| \| Traditional doctor \|  \|  \| \| Clinic \|  \|  \| \| Self-medication \|  \|  \| \| Other \|  \|  \|   43.1. If yes, ¿what type of care did you receive?  44. ¿Have you missed days of work or study after hospitalization? Otherwise? Yes____ No _______  44.1 ¿How many? _____ | | | | 45. Specify how much you had to pay for this care   \| **EXPENDITURE** \| **Hospital or clinic** \| **Private doctor** \| **Health center** \| **Traditional Medical** \| **Self-medication** \| \| --- \| --- \| --- \| --- \| --- \| --- \| \| **Medicines** \|  \|  \|  \|  \|  \| \| **Laboratory exams** \|  \|  \|  \|  \|  \| \| **Diagnostic images (Rx)** \|  \|  \|  \|  \|  \| \| **Consultation** \|  \|  \|  \|  \|  \| \| **Transport** \|  \|  \|  \|  \|  \| \| **Other** \|  \|  \|  \|  \|  \| \|  \|  \|  \|  \|  \|  \| | | | | |  |
| **OBSERVATIONS** | | | | | | | | |  |
