# Supplementary material for: Economic costs of severe seasonal influenza in Colombia, 2017–2019: A multi-center analysis
Source: PLoS One. 2022 Jun 17;17(6):e0270086. doi: 10.1371/journal.pone.0270086 (PMC9205505; doi:10.1371/journal.pone.0270086)
Supplement: S1 Text — (DOCX) [file pone.0270086.s006.docx]

S1 Text. Costing Reporting Checklist

Source: Vaughan K, Ozaltin A, Moi F, Kou Griffiths U, Mallow M, Brenzel L. Reporting gaps in immunization costing studies: Recommendations for improving the practice. Vaccine X. 2020;5:100069

| **Checklist Item** | **Reporting Options**[**^*^**](https://www.ncbi.nlm.nih.gov/pmc/articles/PMC7451807/table/t0015/?report=objectonly#tblfn1) | **Verification** |
| --- | --- | --- |
| ***Study design and scope*** | |  |
| Study type | Example responses: Costing study, economic evaluation, financial planning, budget impact analysis, efficiency analysis, other | “A bottom-up costing study” is declared in study design |
|  | |  |
| Aim of the cost analysis | Free text; may be useful to include a description of the process undergone to arrive at the aim, including stakeholders (target audience) and how they were engaged | The objective of this analysis was to estimate the economic costs of SARI in Colombia, from a third and social perspectives, assessing direct medical and non-medical costs in patients with laboratory-confirmed influenza Colombian in health facilities. |
|  | |  |
| Relevance for health practice and/or policy decisions | Free text; address any relevant parameters of practice and/or policy that informed the conceptualization and design of the study | Second paragraph in the introduction: “To reduce the burden of influenza disease, various strategies have been proposed, including vaccination specific population groups such as children under five, adults over 60, pregnant women, and health workers; however, economic considerations, as detailed cost analysis, are an essential input to effectively guide the formulation of policies for influenza immunization (7). Decision makers, particularly in lower- and middle-income countries, lack economic data to support influenza vaccine policy decisions, then information about both direct and indirect cost impacts due to influenza is needed (8)” |
|  | |  |
| Vaccines costed | Recommended sub-items: Manufacturer, trade name, presentation (doses/vial or pre-filled syringe), packaging and packed volume (cm3/dose)  Schedule of required doses and timing Unit price per dose Total doses delivered at study sites (or other totals used for extrapolation beyond the sample) | We did not evaluate vaccine costs, however the details about the activities and supplies considered in our costing analysis are included in methods section and as supplementary information (S1 Table) |
|  | |  |
| Target population | Recommended sub-items: Age, sex, geographical location, etc. of those targeted for vaccination | In methods: “The study population was all patients admitted by emergency room or outpatient clinic that met the clinical SARI definition” |
|  | |  |
| Coverage level | Recommended sub-items:  Coverage of costed vaccine as percentage of target population at sites For costing of multiple vaccines, overall program coverage | Did not apply for our costing analysis |
| Delivery strategy and sector | Recommended sub-items:  Health facility, outreach/mobile, school, campaign, national immunization days/weeks or child health days/weeks, along with description of the strategies Routine or supplemental immunization activity (SIA)  Public, private or NGO | Not apply for a particular disease costing analysis, in our case only inpatient cases were included. |
|  | |  |
| Study perspective | Example responses: Government, provider, health system, societal, other (may include military, schools, etc.). Free text to define the perspective with clarification on disbursing agents or the ultimate sources of funds | “The present analysis is a partial economic evaluation (16) from the third payer (Colombian Health System) and societal perspectives” is stated |
|  | |  |
| Retrospective costing vs. cost projection | Example responses: Retrospective costing of an existing immunization intervention vs. cost projection for introduction of a new vaccine | We made the costing with both strategies as mentioned in methods: “Patients for the study were selected in two strategies. First one refers to patients who were lab-confirmed influenza patients, during 2017-2019 period, treated in selected centers but that at the time of the start of the study had already been discharged (retrospective recruitment). These patients were selected for the estimation of direct medical costs but did not participate in the estimation of OOPE and indirect costs. Second one refers to patients identified from the start of the study by active search (prospective recruitment) in each included health facility, with suspected SARI consulted and subsequently hospitalized. This second group participated in both costing analysis; direct and OOPE” |
|  | |  |
| Economic vs. financial vs. fiscal cost | Example responses: Economic vs. financial vs. fiscal cost (see [Box 1](https://www.ncbi.nlm.nih.gov/pmc/articles/PMC7451807/#tb1)) | Economic cost stated in the goal and the objectives |
|  | |  |
| Incremental cost? | Yes/no | Not apply, this is a partial economic evaluation |
|  | |  |
| Period type (start-up vs implementation) | Example responses: Introduction/start-up, recurring/ongoing, or both periods included in the costing If reporting introduction/start-up costs, clearly define the start and end points (with dates) for the start-up/introduction period, the included cost categories for each period, and lifetimes and discount rate for annualizing introduction costs | We are costing the entire duration of the disease for any selected case. |
|  | |  |
| Costs included from which level(s) of the health system (i.e. where costs were incurred, though funding may come from different/higher levels) | Example responses: Facility, district, province/region, national, outside the country setting (e.g. international, externally-funded technical assistance). Note if any levels of the health system were explicitly excluded | The costing analysis was performed at individual level (patients with influenza lab-confirmed SARI diagnosis) |
|  | |  |
| ***Service use and measurement*** | |  |
| Cost categories included | Recommended sub-items:  Cost categories included (specify line items separate from activities)  Cost category definitions Justify any cost category exclusions | Categories considered in our costing analysis are included in methods section and as supplementary information (S1 Table) |
|  | |  |
| Sampling frame, methods and size | Recommended sub-items:  Sampling frame Sampling methods Sample size (communities if applicable, facilities, districts, provinces/regions) | Mayor cities and Health facilities selected as a convenience. In each facility “a random sample of at least 50 patients per city” |
|  | |  |
| Timing of data collection | Dates of data collection | We include the clarification “Capture of information was carried out during October to December 2018 and April to June 2019” |
|  | |  |
| Measurement approach for each input | Example responses: Top down/budget or expenditure report extraction, bottom-up/ingredients-approach, or free text | Bottom-up/ingredients-approach implemented as mentioned in Methods |
|  | |  |
| Data source used to measure the units (source, method and allocation/tracing factors to allocate shared resources, such as building space, equipment, human resources, vehicles) | Example responses:  Data sources: Desk/record review (immunization records, expenditure reports, etc.), interviews, observation, post-introduction survey, other Method: structured questionnaires, topic guides, etc. Note how tools were designed, tailored and tested Allocation/tracing factors: Interview, observation, time sheets, floor space, vehicle use logs, other | Frequency of use of activities and supplies was taken for the clinical records and survey applied as mentioned in methods. Instruments are provided as supplemental material |
|  | |  |
| ***Valuation and pricing*** | |  |
| Data sources used for unit prices | Example responses: Desk/record review (budgets, expenditure reports, procurement invoices, etc.), interviews, other For costs incurred outside country setting: note if costs were valued using international or domestic prices | Our methods include a subsection with these clarifications: “**Costs.** Medical billing records were collected to establish direct medical costs. Cost of each item was provided by health facilities in each city. When the cost was not provided by health institution, the tariff manual of the Mandatory Traffic Accident Insurance (SOAT acronym in Spanish) 2018 (20) was consulted. To estimate the medications cost recommendations of the Institute for Health Technology Assessment (IETS acronym in Spanish) were followed, identifying the active principle and the Unique Drug Code from the list of the National Institute of Drug and Food Surveillance (INVIMA in Spanish). It was crossed with the database of the Drug Price Information System (SISMED in Spanish) to obtain the sale price. The weighted average was estimated by number of units reported (21). Oseltamivir® cost was reported by the Ministry of Health (22).” |
|  | |  |
| Methods for valuing volunteer time | Free text | Not included |
|  | |  |
| Methods for valuing donated or subsidized goods | Free text | Not included |
|  | |  |
| Depreciation approach, useful lifetimes and discount rates for capital items | Example responses:  Approach: straight line depreciation, amortization Lifetimes: 10 years, 15 years, etc. and source Discount rates: 3%, 5%, etc. and source | Not capital costs directly included in our analysis, but items as LOS potentially consider it as a price per service |
|  | |  |
| Currency | Recommended sub-items: Currency and year | At the end of Methods section we mention: “All costs were expressed in 2018 US dollar, with an exchange rate of 3,249.75 COP per 1 USD (28)” |
|  | |  |
| Any currency conversions made | Recommended sub-items: Exchange rate, source and year | See above response |
|  | |  |
| Inflation type and rate used | Example responses: Percentage, GDP deflator/CPI, source | Not include because it is a single-point costing estimation |
|  | |  |
| ***Analyzing and presenting results*** | |  |
| Definitions of unit costs used and how they were calculated (numerator and denominator) | Recommended sub-items:  Cost per: dose, capita, person in the target population, fully immunized child, full immunization of a vaccine, other unit costs (such as cost per cubic meter for supply-chain related studies) | The equation is provided to estimate the average cost per patient:  $=\frac{\boldsymbol{\sum(}\boldsymbol{cost}_{\boldsymbol{A}}\boldsymbol{)*(}\boldsymbol{frequency of use}_{\boldsymbol{A}}\boldsymbol{)}}{\boldsymbol{(n)}}$ |
|  | |  |
| Analytical techniques used | Recommended sub-items, if applicable, with free text response:  Aggregation Generalizing costs beyond the data collected either in terms of time or geography (specify time periods used for generalizing costs beyond the time period collected)  Confidence intervals Multivariate statistical methods used to analyze cost functions Statistical methods used to establish differences in unit costs by sub-group Methods for imputing missing data and extent of the problem | Bottom-up costing technique declared.  Equation to aggregate categories of costing and source of information are reported.  Bootstrapping techniques to calculate confidence intervals also mentioned |
|  | |  |
| Any scenarios or assumptions used for cost projections [if applicable] | Free text | Not applicable for our costing analysis |
|  | |  |
| Relevant total and unit costs | Recommended sub-items:  Report total costs with time period noted (e.g. one year)  Report unit costs with and without the vaccine price included and with and without paid human resources Note percentage share of each cost category of the total and unit cost | Only unit cost (cost per patien estimated) because we used the sampling and not the universe of influenza lab-confirmed SARI cases in Colombia. Percentage share by category are reported for direct costs and OOPE in tables 3 and 4 |
|  | |  |
| Any sub-groups or populations analyzed | Free text | Disegregations by key characteristics of patients are reported in table 2 |
|  | |  |
| Sensitivity analysis findings | Free text | Bootstrapping techniques were implemented to derivate the confidence interval of the estimation. It was reported in Data analysis subsection: “Bootstrapping re-sampling techniques were implemented with 50.000 iterations to approximate the actual values of the Gamma distribution of these parameters (24–27). The mean cost of the resources used was estimated by item.” |
|  | |  |
| Possible sources of bias, limitations in the design, analysis, results and conclusion | Free text; include data challenges and analytical techniques used that may bias results and conclusions as well as aspects of the cost estimates that would limit generalizability of results to other contexts | Limitations of the analysis are presented in the last paragraph of the discussion |

* Depending on the Checklist Item, the Reporting Options column may include example response options, recommended reporting sub-items or further prompts to guide reporting.
